# Improving Identification and Child-Focused Collaborative Care for Children of Parents With a Mental Illness in Tyrol, Austria

**DOI:** 10.3389/fpsyt.2019.00233

**Published:** 2019-04-17

**Authors:** Hanna Christiansen, Annette Bauer, Batool Fatima, Melinda Goodyear, Ingunn Olea Lund, Ingrid Zechmeister-Koss, Jean Lillian Paul

**Affiliations:** ^1^Department of Clinical Child and Adolescent Psychology, Philipps University Marburg, Marburg, Germany; ^2^Personal Social Services Research Unit (PSSRU), London School of Economics and Political Science, London, United Kingdom; ^3^Human Development Programme, Aga Khan University, Karachi, Pakistan; ^4^School of Rural Health, Monash University, Melbourne, VIC, Australia; ^5^The Norwegian Institute of Public Health, Oslo, Norway; ^6^Ludwig Boltzmann Institute for Health Technology Assessment, Vienna, Austria; ^7^Mental Health Research Program, The Village, Ludwig Boltzmann Gesellschaft, Innsbruck, Austria; ^8^Division of Psychiatry I, Department of Psychiatry, Psychotherapy and Psychosomatics, Medical University Innsbruck, Innsbruck, Austria

**Keywords:** children of parents with mental illness, practice approach, implementation, participatory co-design, realist approach, symbolic interactionism, open innovation in science

## Abstract

**Background:** Children of parents with a mental illness (COPMI) are more likely to experience negative long-term adversities. However, interventions to support their needs early can significantly enhance adjustment and reduce negative outcomes. Approximately one in four children currently lives with a parent with mental illness worldwide. The lifelong impact for individuals, governments, and broader society is likely to be substantial. There are significant workforce barriers to the early identification of COPMI and addressing their needs, particularly within the adult mental health care system. The current study aims to reduce such barriers and to improve identification of COPMI in the current health care systems.

**Objectives:** The project “The Village” is a multidisciplinary health and social care policy intervention and seeks to improve child development and well-being outcomes for children of parents with a diagnosed mental illness. This will be achieved through the co-development, implementation, and evaluation of a practice approach to the early identification and collaborative care for COPMI, through establishing child-focused support networks. This will be done with open innovation science (OIS) approaches engaging the public in Tyrol, a geographical region of Austria, throughout 4 years. As part of the co-development process, we will work with stakeholders to co-develop the practice approaches based on evidence-based approaches and determine the most appropriate study design to evaluate those, as well as the implementation processes we will undertake.

**Methods:** The project is underpinned by theories from different disciplines (i.e., public health, psychology, sociology, linguistics, economic sciences) as well as drawing on different approaches (i.e., co-development, implementation science, symbolic interactionism, and realist evaluation). It is based on the seven content work packages (WPs): 1) management, 2) focusing on children and methods to understand their “voice,” 3) scoping, 4) co-development, 5) implementation, 6) evaluating the practice approaches, and 7) knowledge dissemination. “Scoping” will involve exploring the existing evidence, practice, and current state of identification and collaborative care in Tyrol, Austria. “Co-development” involves the co-design of practice approaches to identify and support children in partnership with key stakeholders and service providers working in Tyrol. The “implementation” of practice approaches will be based on the results of the co-development phase and will involve working with organizations to develop support strategies that draw on known organizational drivers from the field of implementation science to support the rollout of the practice approaches. In “Evaluation” we will follow principles of a realist approach; this includes developing program theories and logic models for the practice approaches. Those will set out the outcomes hypothesized to achieve and the processes that are expected to lead to those changes. This will refer to changes in children, parents, and practitioners. We expect that the main focus will be on measuring child quality of life and mental health outcomes, and outcomes that are on the path to those (such as social support needs, resilience, mental health literacy, stigma, and help-seeking behavior) as well as costs. The “child voice” WP focuses on children’s perceptions and needs as the importance of “assent” and support of children to develop their own “voice” in health care is increasingly recognized within child health research. The “dissemination” step focuses on reaching a broad public audience of different stakeholders, researchers, and families involved.

**Discussion:** The research project aims to directly improve identification and support of vulnerable children across selected regions in Tyrol, Austria, and by doing so, improve the health and well-being of future generations, through breaking the cycle of intergenerational transfer of adverse childhood experiences.

## Introduction

It is estimated that 50% to 66% of people with serious mental illness are living with one or more children (1), and that approximately 25% of children live with a parent who has a mental illness (2–6). Having a parent with a severe mental illness (SMI) has been associated with adverse child development outcomes, which can have long-lasting effects throughout a child’s life including multiple physical and mental health problems (3, 7–10); lower academic achievement (11, 12); and reduced employment opportunities (13). While most studies on COPMI have been carried out in the United States, United Kingdom, and Australia, there is now also evidence from a German national mental health survey showing that parental mental illness is associated with increased risk of mental health problems in children and adolescents (14). A community study found that children of parents with SMI carry a higher risk of developing mental disorders compared to children of parents with mild to moderate mental illness (3). Thus, children of parents with a mental illness (COPMI) are more likely to present as the next generation of people living with SMI and who will use mental health services (10). Further, studies show the overrepresentation of this population in child community mental health services: 48–79% of the children using community mental health services had a parent with a mental illness (15–17). This transgenerational transmission of mental disorders (TTMD) is associated with high costs: In the United Kingdom, the estimated costs of adverse child impact linked to maternal mental illness during the perinatal period alone is £5.8 billion per year (18, 19). The risks associated with the TTMD could result in impaired parent–child interactions, genetic and pregnancy risks, emotion regulation deficits, individual vulnerabilities such as difficult child temperament, stress reactivity, cognitive skills, as well as social environmental factors such as school/work environments and social support. These risk factors are summarized in the Hosman et al. (20) model of the TTMD, though as yet we know currently of only one study testing this model (21).

The treatment of the parental disorder is associated with improved mental health and well-being in COPMI (22–27), though overall there are only a few studies on such effects (25, 28). These studies typically target the same disorder in children and parents, e.g., depressive symptoms in the offspring of parents with depression. However, such a specific transmission of disorders is not typical, as the outcomes often follow a *multifinality* pathway (20) with children of parents with depression developing various kinds of disorders (10, 29). A meta-analysis with nine studies on the effects of psychological treatment of maternal depression on children’s psychopathology resulted in an overall moderate effect size, with a Hedge’s *g* = .40 (28). An earlier meta-analysis on preventive interventions for COPMI demonstrated a significant relative risk reduction of 40% in the same disorder as their parent, and overall small effect for children’s internalizing (Hedge’s *g* = -.22) and externalizing (Hedge’s *g* = -.16) symptoms (30). The most recent and comprehensive meta-analysis on preventive interventions for COPMI (31) resulted in effect sizes similar to those of Cuijpers et al. (28) for young children, and overall smaller effects for older children that equal those of Siegenthaler et al. (30). Different longitudinal studies on parental anxiety and depressive disorders present heterogeneous effects of parental treatment on children. A 6-year prospective longitudinal study on the effects of parental panic treatment demonstrated that parental treatment is a significant predictor of children’s anxiety symptoms (25). The Sequenced Treatment Alternatives to Relieve Depression (STAR*D) Child study is designed to examine the association between maternal remission from depression and children’s functioning and psychopathology. The study demonstrates differential effects on child psychopathology in early, late, and nonremitting mothers, with early remission being associated with reduced child externalizing problems (27); similar results have been obtained in another large longitudinal study (22). Thus, overall positive parental treatment effects are associated with reduced psychopathology of the children, though changes in important markers such as well-being, or for children that have not developed psychopathology symptoms yet, are missing.

A major barrier for such positive effects of the parental treatment is the lack of identification of children in families with mental illness. A recent review identified only nine studies systematically assessing whether adult mental health patients admitted to either in- or outpatient care had children or not, and revealed a substantial number of patients that were either not asked about parental status or where this was not documented in patient files (32). The same holds for child and adolescent mental health services (CAMHS) that do not systematically assess the health status of the parents of children and youth in treatment (17). Many systemic barriers exist preventing the early identification of COPMI in adult mental health services including a lack of skill and knowledge of practitioners to talk about parenting, lack of collaboration between the different services involved (i.e., adult and child mental health), individualized funding and treatment focused models of care that limit a family focus in treatment, and a reluctance by parents to talk about their children for fear of child removal into care (33–35).

Hence, according to the review by Maciejewski et al. (36), we are currently lacking opportunities for early identification and thus preventive interventions for COPMI due to adult mental health services neglecting the impact of the TTMD by not asking about parents and children. Developing opportunities or practices that help integrate a focus on intergenerational impacts of mental illness in adult mental health in ways that manage these systemic barriers are considered to be essential (37).

Interestingly, other disciplines have realized the importance of such early identification, especially pediatric primary care (38). As up to 15% of mothers and 7% of fathers suffer from postpartum depression (PPD) (39), they suggest PPD screening in pediatric primary care in order to identify and treat parents and to prevent negative PPD impact on the children, as this has been associated with a high risk of developmental delays and behavior problems (38). This study thus further highlights the necessity of strong collaborative care that, in practice, is rare and challenging between relevant disciplines (40, 41). Currently, this lack of collaboration leads to a lack of identification and thus missed prevention opportunities.

Another significant barrier is the current lack of collaboration itself. Current research suggests a lack of collaboration between different sectors that may impact COPMI—such as adult psychiatry/psychotherapy, child and adolescent psychiatry/psychotherapy, youth welfare agencies, pediatrics, gynecology, as well as family and social services. Instead these sectors offer individualized services that neglect the family as a whole, and the opportunity to capitalize on coordinated services is lost (42, 43). Thus, services that are in touch with families where a parent has a mental illness do not connect and miss chances that might improve overall family and especially child functioning and well-being (33, 40). Even in countries that have taken legislative steps to improve identification of children in families with mental illness, such effective collaboration is still scarce (44). As a result, COPMI often remain invisible, and their needs may be unmet, as their “voice” usually remains unheard (45).

Our project “The Village” has been designed to address these barriers to the care of COPMI through utilizing an open innovation approach (46–48) to design and trial evidence-informed practice approaches in adult mental health and other family support services using principles of symbolic interactionism, realist evaluation, participatory co-design, and knowledge of translation to practice implementation strategies from the field of implementation science (for elaboration on those methods, see below).

The current study has two major objectives: in our model region in Tyrol, Austria, we will 1) facilitate identification of COPMI with a *Sensitive Screening* (SENSE); and 2) establish a *Collaborative Village Approach* (CVA) that will enhance the formal and informal support opportunities for children. The project will be carried out with a focus on understanding children’s experiences, challenges, and opportunities to support a child-focused SENSE and CVA, the child’s voice. The project aims to ultimately improve child development and well-being outcomes for children and families.

## Methods

**Figure 1:**
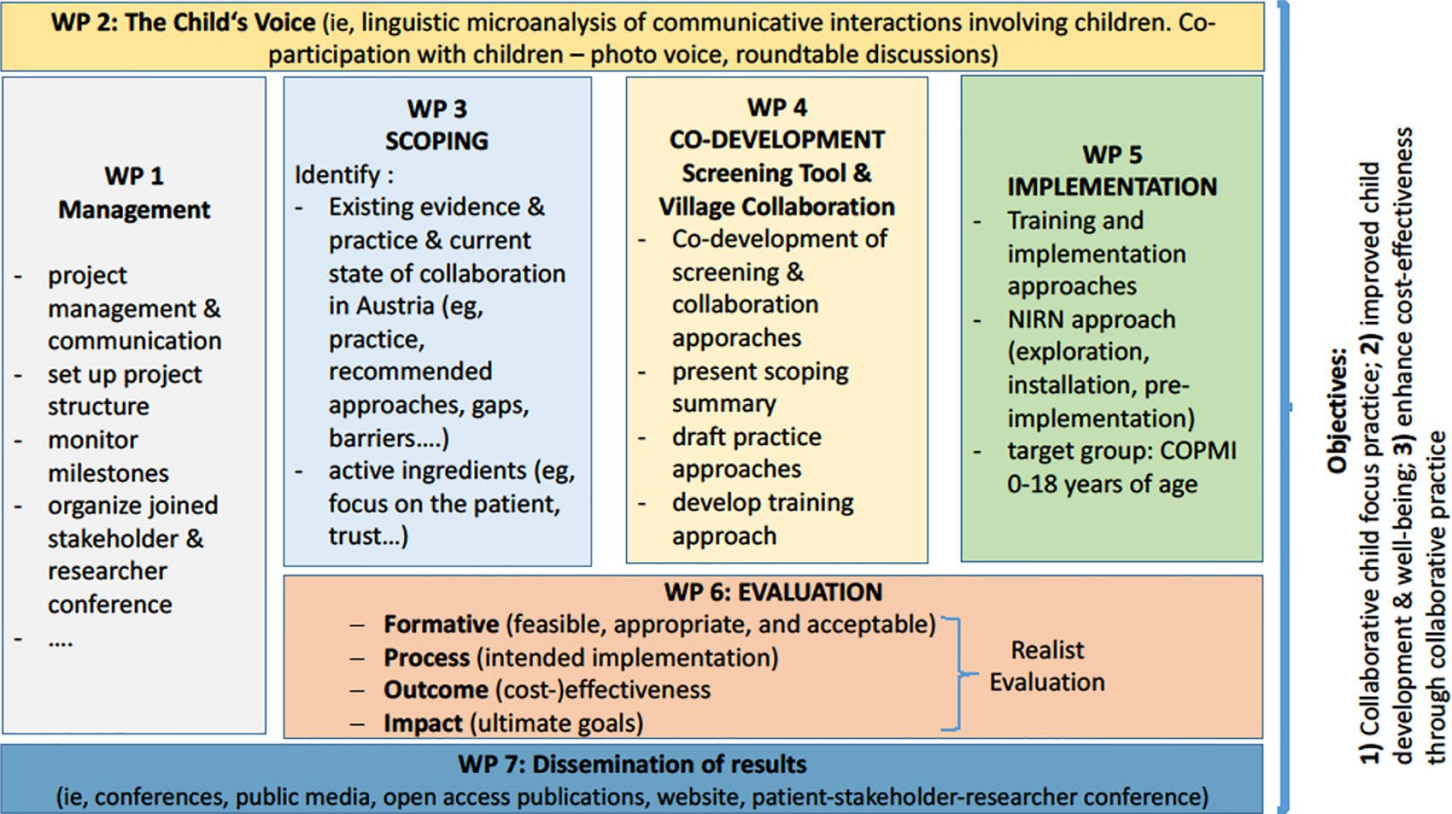
Objectives and work packages (WP) of “The Village.”

The project will be carried through seven work packages to scope, co-design, implement, and evaluate the practice approaches (see **Figure 1**).

### Design and Theoretical Framework

The project is underpinned by the theories from implementation science (49), a realist evaluation approach (50), and symbolic interactionism (51, 52).

Installing any new practice in routine care is difficult. Many gaps exist, particularly in mental health services, between what is known to be effective evidence-based practice and the translation of these practices into routine care (53). To help move beyond a “train and hope” strategy for workforce development, working with ways to address the systemic barriers within and across services that prevent practice change is a particular focus of implementation science, particularly for practice approaches that aim to address the intergenerational impact of mental illness in families (37). According to the National Implementation Research Network, implementation is defined as a specified set of activities or strategies designed to put into practice an evidence-based program or activity into routine use within specific settings (54). The field of implementation science acknowledges the need to focus on the feasibility and acceptability of new “intervention”/practice change, in order to facilitate practice change. The principles of co-development and co-design approaches that aim to work alongside key stakeholders in applied settings to co-create and adapt the evidence-based practice with local contingencies in mind, particularly in complex health environments, are one such approach drawn from implementation science to address the gap from research to practice (55).

A realist approach toward evaluation is a theory-driven type of evaluation that is particularly suitable for the examination of complex programs in the health and social care field. It advocates the investigation of theoretical assumptions underpinning a program and whether those are responsible for changes that may occur. The aim of each realist evaluation is to provide a detailed description and analysis of contexts, assumed causal origins, and mechanisms that, in turn, affect the outcome of an intervention (56). Realist evaluation is based on the theory of critical realism as proposed by Archer et al. (57). According to this underpinning theory, the potential mechanisms of causation reside both in the individual actors as well as in society and are real and present even when not active and may or may not be observable when actualized. Realist evaluation is thus different from traditional evaluations that focus on overall effectiveness in that it provides a basis to describe how and why a complex intervention works or does not work (56). Both empirical qualitative and quantitative data will be used to examine not only whether the practice approaches were (cost-) effective but also possible processes and contextual factors that influenced outcomes (or costs).

The theoretical framework of symbolic interactionism is based upon the epistemology of constructionism and refers to the belief that meaning and knowledge are constructed and maintained through social interactions; there is no one truth to be discovered, but many depending on the way in which the question is asked, approached, and analyzed (51, 52). As such, it is important to continuously gather data and perspectives of multiple stakeholders throughout the Village project that provide feedback into the project design. Such an approach focuses on the importance of language and interactions in shaping how people make sense of themselves and their social world (58). Given the stigma experienced by people and families impacted by mental illness, along with the inherent sensitivities of trying to find and support children in this situation, examining the social and interactional nature of experiences is particularly relevant for this study. Research using symbolic interactionism values multiple data sources, commonly obtained through qualitative approaches, and perspectives in order to continuously build and reflect the social interaction of interest. Aligned with this theory, we undertake a period of exploration (scoping). The co-developed approach also acknowledges the importance of collecting multiple views in order to produce new practices that will more likely fit the situation. Obtaining multiple data sources, such as interviews or observations from different perspectives or media, known as data triangulation, can enhance interpretive rigor, a valuable quality check used within qualitative research.

With an open innovation science (OIS) approach, i.e., the stra­tegic use of the public to increase innovation (46, 47), we will decide together with the relevant stakeholders on the research steps to be taken and consult monthly with a group of “experts by experience” (young adults with a parent with a mental illness). Our project’s use of OIS will be internally assessed by the Open Innovation Centre at the Ludwig Boltzmann Gesellschaft using novel evaluation criteria based on opening up disciplinary boundaries, fostering public engagement in the research process, and establishing new forms of stakeholder interaction and collaboration that lead to interdisciplinary and transdisciplinary research (59).

The processes of this methodological framework as well as their linkage are described in detail in the work packages “scoping,” “co-development,” “implementation,” and “evaluation” (see below).

The other three work packages of the Village describe the overall management, dissemination of research findings, and our central overarching focus of the project on capturing the “child’s voice.”

### Scoping

Drawing on implementation science, in a staged model of organizational change, scoping (known as the exploration stage) is a critical first step needed to understand the contextual factors shaping existing practice and practice challenges that need to be addressed in the implementation of practice approaches for the translation to routine care (60). This initial step will therefore generate the knowledge and conceptual frameworks to be informed and to be tested in the co-development, implementation, and evaluation of the project. This will include summaries of evidence and draft logic models that inform the development of the two practice approaches, SENSE and CVA. Scoping will be supported by the knowledge generated on conceptualizations of the child’s voice (described below).

Evidence will be collected through reviews, and OIS principles such as stakeholder views; sharing and exchanging will be applied to understand and describe 1) current practice, recommended practice, and gaps in relation to the identification of COPMI as well as in relation to current and recommended collaborative care that puts the child at the center of attention (“child’s voice”); 2) the (unmet) needs for COPMI; 3) what works for whom and when, what is cost-effective, and what are the mechanisms (active ingredients); and 4) contextual factors that can inform the development of logic models and the development of approaches.

As this work is underpinned by the theoretical framework of symbolic interactionism (51, 52), it is essential to gather perspectives of multiple stakeholders to inform co-development and implementation.

Particular pieces of work to address the scoping objectives will be conducted, using varied techniques:

- mapping of existing Tyrolean support structures and epidemiological dimensions, and mental health service use;
- semistructured interviews with Tyrolean stakeholders in the community, from services and families, to analyze the current situation of supporting COPMIs, existing needs, and barriers in Tyrol;
- mapping of key topics in the COPMI research field to identify what topics are studied together and which are not (and what the current research focus is) using co-word analysis, bibliometric coupling, and co-citation analysis;
- systematic review identifying facilitators and barriers in identification and support provision for COPMI;
- interviews with international experts who have attempted to change adult mental health care to be family-focused in order to improve identification of children at risk of mental illness;
- review of collaborative practice within the COPMI setting to identify the “active ingredients” or what has been shown to work.

The results from the Tyrolean mapping and situational analysis are currently summarized in a separate paper (61). Our preliminary evidence suggests that a variety of services are available both for parents who have a mental illness as well as for families and individual family members. However, services do not address the needs of COPMIs specifically and lack coordination across sectors. Findings further indicate that there is awareness of the problems related to COPMIs at all levels, but there is a lack of installed support processes to meet their needs.

Finally, initial logic models will be drafted in line with a realist approach (50). This work will inform the co-development process where the logic model will be developed further and agreed upon with stakeholders, and the co-creation of new practice approaches with stakeholders that draw on the evidence base for SENSE and CVA.

### Co-development

One key element of this research project is that it follows OIS principles (46). This includes the co-development of the practice approaches for SENSE and CVA (informed by knowledge generated during the scoping in the form of initial logic models and the research literature) with local stakeholders and the training and implementation support for the application of these co-created practice approaches into routine care. Furthermore, stakeholders will be involved in decisions about key indicators and (outcome) measures and study design for the evaluation of the practice approaches.

**Figure 2:**
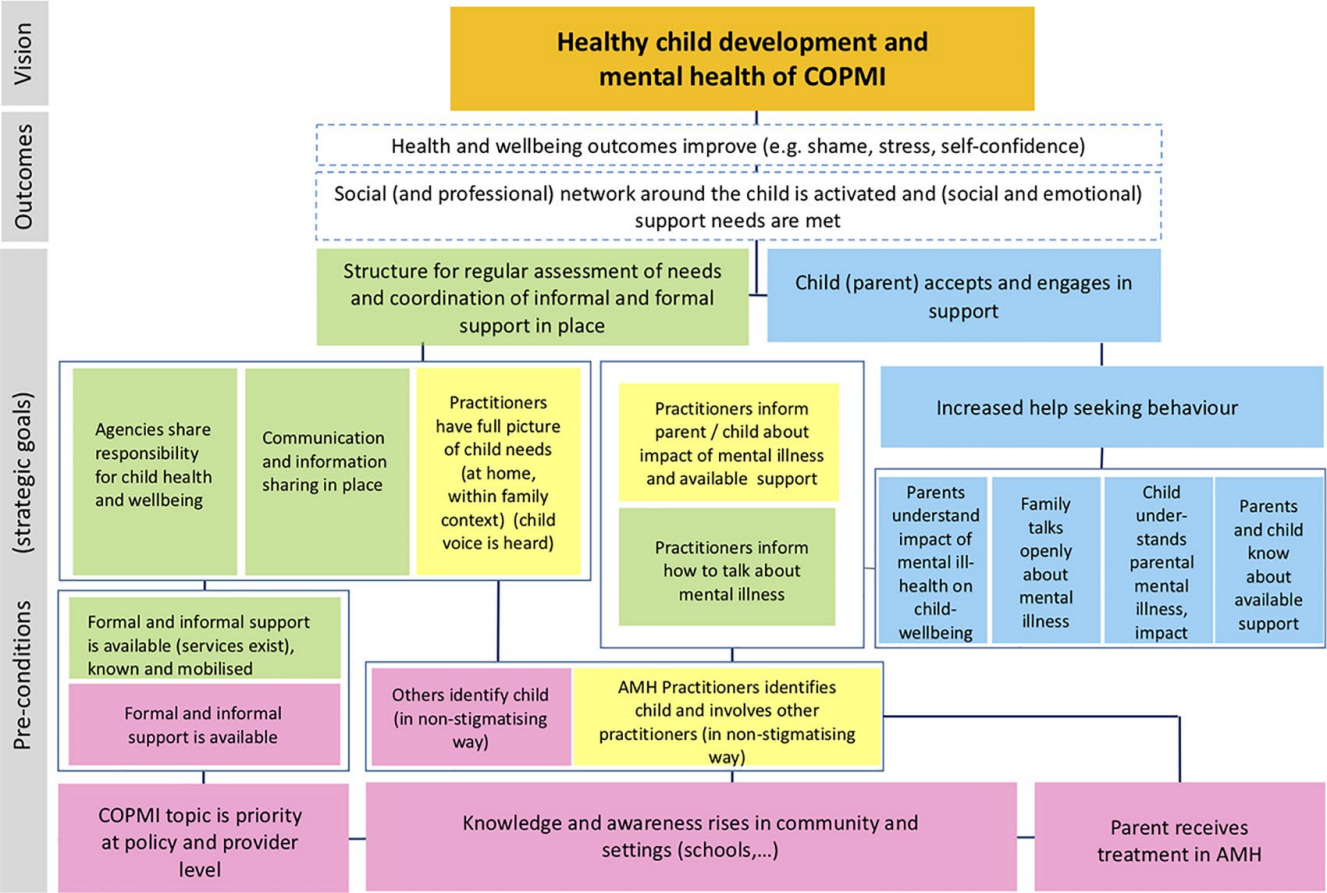
Preliminary theory of change. Green boxes refer to the CVA approach; yellow boxes to SENSE; pink boxes refer to general elements; and blue ones to the family. AMH = Adult Mental Health; COPMI = Children of Parents with a Mental Illness.

A series of design workshops with stakeholders representing key service providers from the Tyrol region will be held monthly over a 6-month period to draw on the evidence and logic models developed in the earlier stages and existing practice wisdom of key stakeholders to co-develop the components of the two practice approaches (SENSE and CVA). Key stakeholders include leadership representation from psychiatric/psychotherapeutic and social care services across the region supporting adults and/or children, with a mix of professional background, and include people with lived experience of parenting with a mental illness or being an adult child of a parent with mental illness. Consultation will also occur with COPMI (the beneficiaries) acting in an advisory capacity. A training concept and training materials as well as feasibility and implementation indicators will also be prepared with the input of key stakeholders to support the implementation of the practice approaches in their respective contexts. To date, four out of six workshops have been held and draft models for SENSE and CVA for the Tyrolean region are already available. A preliminary Theory of Change model was presented to the workshop participants, which was based upon initial logic models, literature, and data collected during the scoping phase (see **Figure 2**). All workshops will be recorded, transcribed, and analyzed. Additional data will be collected *via* participant observation and by holding a focus group after the final workshop. Qualitative methods will be applied to analyze the data collected to measure the impact and process undertaken during this co-development phase. Core themes will be identified (based on a content analysis), clustered, and critically analyzed (62).

### Implementation of the Practice Approaches (SENSE and CVA)

The objective of this step of the project is to implement the key practice approaches and position the evaluation of the impact into the applied settings decided on in the co-development stage. This stage of the project will draw on best practice evidence using known facilitative implementation drivers (49) to support organizational change to embed the 1) SENSE and 2) CVA approaches into selected Tyrolian study sites. Implementation drivers (also known as core components) are a key focus of implementation of the new practice approaches to ensure that the project

- develops, improves, and sustains the ability of selected sites to deliver the new practice approaches and
- creates an enabling positive organizational context that can support and sustain the operation of the new practice over time (49).

Implementation drivers are activities and processes for building the capacity and infrastructure of an organization to influence a program’s success and are the “engine of change” needed to initiate and support changes to achieve positive outcomes for children, families, and community (49).

Underpinning these drivers is the level of Program Acceptance and Program Buy-in by staff, including managers and leadership, to the new practice approaches. When attention and action are paid to these key drivers, we are much more likely to see the practice approaches implemented as designed, resulting in change to practice and therefore improvements in family outcomes.

The evidence-based implementation framework will inform the project from the scoping through to the evaluation phase. Implementation is concerned with the use of strategies to adopt, integrate, and use evidence-based interventions/practices to change practice patterns within specific settings. This approach will utilize the NIRN implementation framework developed by the National Implementation Research Network (49) (exploration, installation, implementation), and undertake a series of activities across the entire project that will attempt to understand the context of implementation (the scoping of current practice and existing barriers at selected sites), to design a feasible approach that fits to the context (the co-development approach), to help prepare the environment, to train dedicated staff, and to coach the practice of the approach at selected sites. This implementation phase will also monitor the uptake and address issues with the on-going implementation of the approach at sites participating in the evaluation.

### Evaluation

The main objective of the evaluation is to generate knowledge as to whether SENSE and CVA were implemented successfully and whether they were (cost-) effective. More specifically, the objective is to understand feasibility and acceptability of the practice changes, with a focus on the co-developed nature of the process, and on mechanisms and contextual factors; changes in outcomes (i.e., child quality of life and psychopathology, or outcomes that are expected to be on the path to child quality of life and mental health improvements, such as social support, mental health literacy, stigma, confidence); and changes in service use for COPMI and their family. We will also seek to capture impact at the wider system level. We will need to agree on outcomes with stakeholders, but at the moment, according to our current theory of change (**Figure 2**), we will measure mental health literacy, stigma, help seeking, quality of life, and psychopathology.

Evaluation of SENSE and CVA will follow principles of the realist approach, which is particularly suitable for complex interventions, and takes into account the mechanisms and conditions for change (50). Generation of the logic models will inform the framework of evaluation. Planning for process evaluation will start during scoping and co-development and inform modifications before full implementation of the practice approaches. Indicators will include a number of professionals trained, parents screened and children identified, children or families referred, collaborative “Village” meetings held, as well as awareness and behavior change in professionals. Knowledge generated about program theories (e.g., in the form of logic models) during scoping and co-development will inform the evaluation of outcomes and costs. Outcomes, resource use, as well as views and experiences will be captured at baseline and 6- and 12-month follow-up (i.e., after baseline) to assess changes for children and parents. Evaluation parameters and measures will be agreed upon during the scoping and co-developing phase. Personnel at study sites will be trained in the recruitment of study participants and practice approaches during the implementation phase. The cost-effectiveness analysis (in the form of cost-consequences analysis) will examine direct and indirect costs from a public sector as well as a societal perspective, and compare them against outcomes. Statistical analysis will be carried out to examine the causality of SENSE or CVA on outcomes and service use (and costs). The choice of statistical method will depend on the final choice of the study design.

### Understanding the “Child’s Voice”

Each of the steps outlined above will be influenced by an overarching focus on capturing the child’s voice in the research undertaken. The project will explore and identify the concept of the “child’s voice” in COPMI health settings, and knowledge produced will inform the overall research process, particularly in the co-development, implementation, and evaluation. This part of the project will identify how children currently, and wish to, participate in discussions about their needs, from the perspective of the child, service providers, and parents. It will uphold the project’s underpinning philosophy to hold the child at the center of their care, and to listen and support them in identifying their own formal and informal support network. It will provide opportunities to educate professionals in the implementation step in appropriate ways to engage and support COPMI to become more communicatively active in conversations with adults about their needs in the evaluation step.

Focus groups and interviews primarily conducted for scoping and co-development will include questions that will address the topic of “child’s voice” and communication between children and service providers [c.f. Ref. (63) for an example of methodological approaches]. This work will support the International Charter for Human Values in Healthcare—Compassion, Respect for Persons, Commitment to Integrity and Ethical Practice, Commitment to Excellence, and Justice in Healthcare. It will also draw upon research regarding health care communication with vulnerable people, such as those with disabilities or individuals from migrant populations, in which traditional patient–doctor power balances are amplified, as is the case with doctor–child interactions.

Through close analysis of naturally occurring health care interactions and analysis of stakeholder perspectives, this work aims to understand and improve the nature and possibilities of children’s interactive participation in conversations about their needs and concerns. This work spans across the project and makes use of and informs data collected in the other work packages. A series of sociological and linguistic micro-analyses will be performed across collected data sets. Additional data collection will include (audio or video) recordings of health care encounters with children, as determined through consultation with stakeholders in scoping and co-development. This part has already started as we record communications between health care professionals and children. A total of 40 interactions will be recorded. For comparison purposes, those include interactions between health care professionals and children in need of psychotherapeutic/psychiatric treatment as well as interactions between adult health care professionals that treat the parents and their children. We will also investigate current practice (i.e., case meetings) to determine how others do and can speak for the child if they are unable or not present.

The communicative effectiveness of SENSE and CVA to listen to the child’s voice and address their needs will be evaluated once this phase of the project starts through recording observations and will also inform and utilize the evaluation. Discourse analytic techniques will be applied, such as Conversation Analysis (CA), pragmatics, and interactional sociolinguistics, to analyze interviews, focus groups, and naturally occurring interactions, which will provide opportunities to educate professionals in this field.

### Dissemination

The objectives of the dissemination focus are based on OIS (46–48): (1) to gain ownership and buy-in for the research among stakeholders throughout the different research phases and (2) to achieve impact at an individual, community, regional, and (inter-)national level. This includes impact in regard to research, practice, and policy. All communication and dissemination will be designed sensitively, with the aim to reduce stigma as a relevant barrier to the realization of the project (45).

Communication and dissemination will be an ongoing process from start to end of the research. This includes identifying organizations and representatives of those who will be part of the project; relevant stakeholders will be recruited into advisory and steering groups; a series of open-day forums will be organized throughout the project to which a wide range of stakeholders will be invited. This will include families, service managers, politicians, researchers, commissioners, and practitioners (including clinicians, social workers, school teachers). We inform about upcoming events and provide information and material for all events already having taken place (https://village.lbg.ac.at/news). Events will inform stakeholders about the research and include educational components; for example, keynote speakers might talk about the latest evidence and good practice; policymakers and influencers (including patient and professional associations) will be engaged; partnerships will be built and managed with organizations and projects that have similar aims and work with similar target groups such as early interventions that target families with newborns to support them and promote healthy child development; and formal collaboration agreements will be established. Our first two formal events for stakeholders were held in Innsbruck in June and September 2018, including the project inauguration (“Kick off”) and the first of our annual “General Assemblies.” These events are an opportunity to present updates to our official and unofficial stakeholders, receive feedback on design and findings, promote the project to policymakers and people working with children in Tyrol, and raise awareness in a sensitive approach that aims to reduce stigma surrounding mental health. Ongoing information is made available *via* our Village research project website (http://village.lbg.ac.at). A range of innovative communication tools (e.g., video, infographics; see: https://www.youtube.com/watch?time_continue=64&v=XHBv7ebFkWU) will be developed, which will be disseminated together with research reports throughout the project to promote the research aims, methods, and findings. Peer-reviewed papers will be produced and published in scientific journals, and a joint researcher–stakeholder conference is planned at the end of the project to communicate findings to the general public.

### Analytical Strategy

As outlined in our theoretical framework, as well as in the detailed descriptions of the different work packages, the Village intervention differs substantially from traditional RCTs or other traditional scientific approaches. Although this may bring several risks to the project, it provides a significant opportunity to understand and implement practice approaches that address the local needs in Tyrol. Currently, in Austria, as well as the other German-speaking countries Switzerland and Germany, there are no sustainable structures for COPMI. The Village aims at understanding the currently available structures and how to improve and connect them in order to achieve an improved identification of COPMI with our SENSE approach, as well as improved care with the CVA approach. For this, we will use the tools provided within our theoretical approaches. As those strongly depend on OIS and stakeholder participation, those will be developed and decided on interactively and in collaboration. We thus cannot provide a fixed analytical strategy, as this is work in progress. We have, however, done some preliminary sample size calculations to determine numbers needed to treat to observe change (calculations were performed using the “n4means” function from the R package “CRTSize”; http://r-project.org). As outlined in the Introduction, we can expect small to moderate effects (31). As there are currently no studies on COPMI and the SENSE and CVA approaches, we assume at best small effects. Depending on the number of clusters (i.e., participating sites), the number of participants to be included varies largely. For instance, if we assume a small effect (Cohen’s d = 0.1) of our CVA approach as well as clusters participating in Tyrol with an intraclass correlation of 0.01 with α = 0.05 (two-sided), 1-β = 0.8 in a parallel-group cluster-randomized trial, we would need 3,420 participants in 342 clusters (cluster size m = 10). **Tables 1** to **3** provide information on the number of participants to be included for different cluster sizes. However, those numbers depend on whom we will include at what stage with what expected effect. They are thus currently only an orientation. The same holds true for potential outcome measures. There are measurements often used in the research of COPMI, such as the psychopathology screening instrument, the Strengths and Difficulties Questionnaire (3), or measures on child-well-being (64), but based on our theoretical framework and as laid out above, we decided to actively involve the relevant stakeholders in this process and give them a voice in our research.

**Table 1:** Required total sample size of clusters and patients to demonstrate an effect of Cohen’s d = 0.1/0.2, assuming an **ICC of 0.1** in a parallel-group cluster-randomized trial with respect to cluster size m.

Cluster size m	Cohen’s d = 0.1		Cohen’s d = 0.2	
	Number of required clusters	Number of required patients	Number of required clusters	Number of required patients
10	596	5,960	150	1,500
20	456	9,120	114	2,280
50	370	18,500	92	4,600
100	342	34,200	86	8,600
500	320	160,000	80	40,000
1,000	316	316,000	80	80,000
2,000	316	632,000	78	156,000
5,000	314	1,570,000	78	390,000

α = 0.05 (two-sided), 1-β = 0.8, ICC = 0.1.

**Table 2:** Required total sample size of clusters and patients to demonstrate an effect of Cohen’s d = 0.1/0.2, assuming an **ICC of 0.05** in a parallel-group cluster-randomized trial with respect to cluster size m.

Cluster size m	Cohen’s d = 0.1		Cohen’s d = 0.2	
	Number of required clusters	Number of required patients	Number of required clusters	Number of required patients
10	456	4,560	114	1,140
20	306	6,120	76	1,520
50	216	10,800	56	2,800
100	186	18,600	48	4,800
500	162	81,000	42	21,000
1,000	160	160,000	42	42,000
2,000	158	316,000	42	84,000
5,000	158	790,000	42	210,000

α = 0.05 (two-sided), 1-β = 0.8, ICC = 0.05.

**Table 3:** Required total sample size of clusters and patients to demonstrate an effect of Cohen’s d = 0.1/0.2, assuming an **ICC of 0.01** in a parallel-group cluster-randomized trial with respect to cluster size m.

Cluster size m	Cohen’s d = 0.1		Cohen’s d = 0.2	
	Number of required clusters	Number of required patients	Number of required clusters	Number of required patients
10	342	3,420	86	860
20	186	3,720	48	960
50	94	4,700	26	1,300
100	62	6,200	18	1,800
500	40	20,000	12	6,000
1,000	36	36,000	10	10,000
2,000	34	68,000	10	20,000
5,000	34	170,000	10	50,000

α = 0.05 (two-sided), 1-β = 0.8, ICC = 0.01. Calculations were performed using the “n4means” function from the R package “CRTSize” (http://r-project.org).

Potential primary and secondary outcome measures might include well-being and psychopathology outcomes for children, improvements in family functioning and social support, and process measures of feasibility and acceptability of the new practice approaches in chosen sites. Barriers and facilitators to service change will also be an important indicator of the co-development and implementation processes of this study. We will also have a focus on measurement of the co-development process and satisfaction with outcomes achieved, as well as documenting the implementation drivers and program adaptation needed to translate the co-designed practice approaches into routine care.

For all activities, we ensure performance according to the Declaration of Helsinki and its later amendments and will obtain approval from the relevant Human Research Ethics Committees (see Ethics Statement). Within the scoping project, we have already performed interviews with relevant stakeholders that are currently analyzed. We have obtained ethical approval for the local scoping interviews and development process measures from the Human Research Ethics Committee of Monash University Melbourne, Australia. In addition, internal approval from the London School of Economics, London, UK for the international interviews is undertaken as part of the scoping stage. Once the recruitment of participants starts, we will beforehand obtain ethical approval from Innsbruck Medical University to ensure accordance with national Austrian guidelines.

### Implications

COPMI are a highly vulnerable group that are still neglected in the health setting in general and the (adult) mental health setting specifically (15, 17, 32) and thus prone to the TTMD (20).

“The Village” aims to change the invisibility of this vulnerable group due to lack of identification and collaborative care in the model region of Tyrol, Austria. To achieve this, we follow a robust collaborative approach based on child voice and OIS principles involving beneficiaries, relevant stakeholders, and policymakers as outlined in the different phases of the project. In the end, this project will have informed and instigated a low-threshold, nonstigmatizing collaborative care system for COPMI in Tyrol, Austria, which efficiently and effectively integrates services and support from the perspective of the child. Accordingly, practitioners and other front-line staff working with children and adults will know how to sensitively identify families with mental illnesses, and how to carry out sensitive screenings (SENSE) to identify COPMI. They will know when additional services are required. If so, they will be able to put the child’s voice at the center and carry out sensitive, low-threshold interventions, based on the child’s and family’s needs. This is expected to improve child development and quality-of-life outcomes and reduce the risk for the development of mental illness in the children themselves, promoting children’s quality of life (65–67). Further, such a best-practice model will be capacity building, providing the evidence for upscaling the practice approaches to other regions of Austria and countries with similar practice challenges. A range of materials about the evidence-informed approaches (SENSE, CVA), and the resource impact of scaling those up, as well as implementation and dissemination strategies will have been made available.

*Impact on service users*: The project seeks to improve child development and quality of life for a population that is often invisible, i.e., COPMI are affected by multiple disadvantages, and often not known to services until presenting with their own problems later during child- or adulthood (42, 43). The project will improve the child’s situation by providing access to a strengthened social support network that supports their emotional, practical, and social needs (45). Although not a specific aim of the project, children will benefit from destigmatized services that put the child at the center of support. This will likely lead to sustainable changes in the child’s life and have positive effects on long-term outcomes that reach into adulthood, such as school performance, employment, and mental health.

*Impact on policy, practice, and research*: The project will test an innovative whole-system approach in an Austrian region. Based on the findings, a training approach, tools, and implementation support package will be developed that (if the project proves cost-effective) would allow scaling up the approach at a national level and may assist practice internationally that seeks to provide holistic care to COPMI. The evaluation can inform a resource impact analysis, which will provide an estimate of the costs to the government (and society) if the approach was to be rolled out more widely. Findings will be disseminated through a number of research outputs. The use of the stakeholders and the media to promote the project will be maximized throughout the project using an OIS design, to increase awareness of the importance of supporting COPMI and ways that this can be done. The message will be that many people in the community can support COPMI. By working with stakeholders, such as patient associations, local community leaders, and health and social care professionals, throughout the entire research project period, knowledge about mental illness in parents and its impact on child health and development will be disseminated. Throughout and toward the end of the project, project stakeholders will be invited to knowledge exchange events, where project progress and results are presented in informal, accessible language.

Through the co-developed research process, the capacity of stakeholders will be built to improve not only their collaborative- and child-centered approaches, but also their evaluation of practice. This includes an increased capacity to collect and analyze data on process, costs, and outcomes to reflect on their practice approaches. This can inform a long-term approach to generating evidence in this area.

*Economic impact*: Economic analysis will estimate the cost-effectiveness from the perspective of government and society. This will include the costs of informal care that occur when children look after their mentally ill parents, as well as other costs that should be included when planning public services (e.g., out-of-pocket expenditure to families and potential long-term costs if children are removed). In Austria, knowledge of the (cost-) effectiveness is currently not well utilized to inform the planning of services. The research team will work with policy stakeholders and support them in translating the research findings into practice.

### Methodological Considerations

“The Village” is a multidimensional project that involves a range of potential risks. Examples of risks include not finding appropriate study sites; too many tasks for the 4-year time period; the unwillingness of stakeholders to be engaged; lack of practice change and fidelity in delivery; and delivering a local project through a multinational team. However, the project includes significant preparatory steps to scope potential study sites, to engage key stakeholders, to co-develop feasible practice approaches, and to use the latest thinking in implementation science to address these potential risks. The project officially started in February 2018 with an initial kick-off event in June 2018. For this kick-off, people with lived experience as well as the range of relevant stakeholders were invited and actively participated in the event. This resulted in positive feedback (https://science.orf.at/stories/2921593/), already suggestive of a high acceptance of and high involvement in “The Village” in Tyrol, Austria.

Finally, the research team will take substantial steps in planning for early identification and mitigation of risks and has indeed started taking some of those steps.

## Conclusion

The research project aims to directly improve identification and support of vulnerable children across selected regions in Austria, and by doing so, improve the health and well-being of future Austrian generations while breaking the cycle of intergenerational transfer of adverse childhood experiences. The described project underwent a rigorous public review process (https://ois.lbg.ac.at/en/methods-projects/crowdsourcing-research-questions-in-science) with different stakeholders as well as people with lived experience and scientific experts of the field having reviewed our proposal (https://ois.lbg.ac.at/en/methods-projects/ideas-lab). This ensures high acceptance and most likely high probability of implementation of our approach, a highly relevant fact for sustained impact. Further, the research group is genuinely interdisciplinary with researchers from public health, (clinical) psychology, economics, linguistics, and implementation sciences thus combining not only different expertise but also perspectives that are crucial concerning cooperation and collaboration. We believe that the research findings from the described public health intervention will also be relevant for health care providers and policymakers in other countries, and the international research community.

## Ethics Statement

The study is performed according to the Declaration of Helsinki and its later amendments and was approved by the Human Research Ethics Committee of Monash University Melbourne, Australia, in addition to internal approval from the London School of Economics, London, UK. Local human research ethics in Austria will be obtained as appropriate. Written informed consent will be obtained from all study participants. If participants are underage, they will be informed about the study and their written informed consent will be accompanied by the written informed consent of their legal guardians.

## Author Contributions

HC wrote a first draft of the manuscript. AB, BF, MG, IL, IZ, and JP reviewed and commented the version. HC and JP revised the commented version, which was reviewed again by all coauthors.

## Funding

“The Village” is a research project funded by the Austrian Federal Ministry of Health–Science and Research through the Open Innovation in Science Centre at the Ludwig Boltzmann Gesellschaft GmbH, hosted at the Medical University of Innsbruck, with the collaboration of Co-Investigator institutions.

## Conflict of Interest Statement

The authors declare that the research was conducted in the absence of any commercial or financial relationships that could be construed as a potential conflict of interest.
